# miR-126-3p-loaded small extracellular vesicles secreted by urine-derived stem cells released from a phototriggered imine crosslink hydrogel could enhance vaginal epithelization after vaginoplasty

**DOI:** 10.1186/s13287-022-03003-x

**Published:** 2022-07-23

**Authors:** Yiyun Xu, Yu Qiu, Qiuning Lin, Chengsheng Huang, Jie Li, Liqi Chen, Zhuowei Xue, Qingkai Wu, Yang Wang

**Affiliations:** 1 Department of Obstetrics and Gynecology, Shanghai Jiao Tong University Affiliated Sixth People’s Hospital, Shanghai, 200233 China; 2 School of Biomedical Engineering, Shanghai Jiao Tong University, Shanghai, 200240 China; 3 Institute of Microsurgery On Extremities, Shanghai Jiao Tong University Affiliated Sixth People’s Hospital, Shanghai, 200233 China

**Keywords:** Extracellular vesicles, Stem cells, Hydrogels, Mayer–Rokitansky–Küster–Hauser syndrome, MicroRNA

## Abstract

**Background:**

Due to the large area and deep width of the artificial neovagina after vaginoplasty, it takes a considerable amount of time to achieve complete epithelization of the neovagina. Currently, the clinical therapies for vaginal epithelization after vaginoplasty are still dissatisfactory. Recent studies showed that small extracellular vesicles (sEVs) derived from stem cells could accelerate wound epithelization. The sustained release of sEVs from optimized hydrogels may be a promising strategy to accelerate vaginal epithelization after vaginoplasty.

**Methods:**

The efficacy of phototriggered imine crosslink hydrogels (piGEL) containing sEVs derived from human urine-derived stem cells (hUSC-sEVs, piGEL-sEVs) on vaginal mucosa defects in rabbits was assessed by wound closure rates, histological analysis and immunofluorescence staining analysis. Cell counting kit-8, 5-ethynyl-2′-deoxyuridine and scratch wound assays were performed to assess the effects of hUSC-sEVs on the proliferation and migration ability of vaginal epithelial cells (VK2/E6E7). Quantitative real-time polymerase chain reaction (qRT-PCR) was carried out to test the expression of epithelial differentiation markers in VK2 cells. Moreover, a microRNA (miRNA) microarray was used to find hUSC-sEVs-specific miRNAs that potentially affected the proliferation, migration and differentiation ability of VK2 cells.

**Results:**

The in vitro release profile revealed that the piGEL could ensure sustained release of hUSC-sEVs. The in vivo results showed that piGEL-sEVs effectively promoted epithelization and angiogenesis of vaginal mucosa defects in rabbits. According to miRNA microarray and qRT-PCR results, miR-126-3p might be the crucial molecule among the various miRNAs contained in hUSC-sEVs. The data showed that hUSC-sEVs promoted the migration and differentiation of VK2 cells by delivering miR-126-3p to suppress the expression of Spred1 and PIK3R2, thereby activating the ERK1/2 and ATK signaling pathways.

**Conclusion:**

The results indicated that piGEL-sEVs could be a novel promising approach for enhancing the epithelization of the neovagina after vaginoplasty and provided useful data for understanding the underlying mechanism of the effect of hUSC-sEVs on epithelization.

**Supplementary Information:**

The online version contains supplementary material available at 10.1186/s13287-022-03003-x.

## Introduction

Mayer–Rokitansky–Küster–Hauser (MRKH) syndrome is the main reason for congenital absence of the vagina characterized by aplasia of the uterus and upper part of the vagina and normal secondary sexual characteristics, with a prevalence of 1 in 5000 live female births [1]. Vaginoplasty is a common surgery for individuals with congenital absence of the vagina [2, 3]. Due to deep and large wound defects after vaginoplasty, it takes a considerable amount of time to achieve complete epithelization of the neovagina. Moreover, patients require long-term use of a vaginal mold. Therefore, more efficient strategies to accelerate vaginal epithelization after vaginoplasty are desperately needed.

Mesenchymal stem cells (MSCs) have gained significant attention with regard to their role in tissue repair and regeneration [4, 5]. Recent studies have reported that umbilical cord MSCs (UC-MSCs) are a new tool for vaginal tissue regeneration after partial vaginectomy [6], and human urine-derived stem cells (hUSCs) display classical features of MSCs, such as multipotential differentiation potential and self-renewal ability [7, 8]. Autologous hUSCs have the advantages of sufficient availability from tissue sources, ease of collection, and cost-effective isolation methods [9]. In addition, hUSCs are highly homologous within the urogenital system during embryonic development. Hence, we proposed that hUSCs are suitable for tissue regeneration and functional recovery in the vagina.

Small extracellular vesicles (sEVs) derived from MSCs can be used as an alternative MSC-based therapy and may be a promising cell-free therapy in regenerative medicine [10]. sEVs secreted by the paracrine pathway of cells are nanoparticles comprising a lipid bilayer membrane enclosing an abundance of proteins, mRNAs, microRNAs (miRNAs) and cytokines. Although the exact mechanisms of these constituents remain unclear, miRNAs seem to play key roles in exosome-mediated therapeutic effects [11]. Recent evidence has suggested that sEVs derived from urine-derived stem cells (USC-sEVs) can exert therapeutic effects by transferring miRNAs to recipient cells and regulating the associated molecular mechanism. For example, USC-sEVs overexpressing miR-16-5p could promote proliferation and inhibit the apoptosis of podocytes by inhibiting VEGFA [12]. However, it's unclear whether human USC-sEVs (hUSC-sEVs) promote epithelization of the vagina by transferring exosomal miRNAs and then affect the related signaling pathways.

Recently, hydrogels have been widely used as biomaterials in tissue repair and regeneration due to their good biocompatibility, biodegradability, reticular crossing structure and so on [13]. Most hydrogels adhere to tissue by physical interpenetration, but this often manifests as poor bonding strength with tissue. In our previous study, a phototriggered imine crosslink hydrogel (piGEL) was developed with excellent properties as a biomaterial due to its UV crosslinked properties, in situ photogelation characteristics, strong tissue integration ability and short gelation time [14]. To prolong the retention time of sEVs on the surface of the wound, we packaged hUSC-sEVs into piGEL, which could guarantee the sustained release of hUSC-sEVs over a period of time. Hence, piGEL-sEVs have been speculated to be a potential therapeutic approach for tissue repair after vaginoplasty.

In this study, we observed the therapeutic effect of piGEL-sEVs on vaginal mucosa defects in an in vivo rabbit model. We also performed in vitro experiments whereby hUSC-sEVs could enhance the migration and differentiation of VK2 cells through enriched exosomal microRNAs.

## Methods

### Cell culture

Vaginal epithelial cells (VK2/E6E7) were purchased from American Type Culture Collection (ATCC, USA). VK2 cells were cultured in Defined Keratinocyte-serum free medium (KSFM, Gibco, USA) at 37 °C in a thermal incubator under 5% CO_2_.

### Isolation and identification of hUSCs

Human urine samples (200 mL per sample) were obtained from healthy young women who provided consent. All procedures were approved by the Ethical Review Board of Shanghai Jiao Tong University Affiliated Sixth People’s Hospital. hUSCs were isolated from fresh urine samples as described in our previous studies [15, 16].

The characteristic surface proteins of hUSCs were detected by a CytoFLEX flow cytometer (Beckman Coulter Life Science, USA). The suspended cells were incubated with monoclonal antibodies (BD Biosciences, USA); the protein targets and corresponding conjugated fluorophores were as follows: CD29-PE (1:100, 561,795), CD34-APC (1:100, 560,940), CD44-FITC (1:100, 560,977), CD73-PE (1:100, 560,847), CD133-PE (1:100, 566,593) or HLA-DR-PE (1:100, 560,943). The results were analyzed with FlowJo X software (TreeStar Inc., USA).

### Isolation and identification of sEVs

As described in our previous studies, hUSC-sEVs were isolated by differential centrifugation and ultracentrifugation [17, 18]. First, to remove dead cells and cell debris, the conditioned medium supernatants were subjected to a series of low-speed centrifugation steps (300 × g for 10 min, 2000 × g for 30 min, 10000 × g for 60 min) followed by filtration through a 0.22 µm sterilized filter (Millipore, Germany). Then, to collect sEVs, the supernatants underwent high-speed ultracentrifugation for 70 min at 100000 × g twice. Finally, the collected sEVs were resuspended in phosphate-buffered saline (PBS, Gibco, USA) and stored at − 80 °C for subsequent experiments. The size distribution and particle concentration of hUSC-sEVs were measured with a nanoflow cytometer (N30 Nanoflow Analyzer, China) as previously described [19]. The morphology of hUSC-sEVs was assessed by transmission electron microscopy (TEM, Hitachi H-7650, Japan).

### Western blot analysis

The proteins extracted from sEVs and cells were separated by sodium dodecyl sulfate–polyacrylamide gel electrophoresis (SDS-PAGE, EpiZyme, China) through 10% gels and transferred to 0.22 μm polyvinylidene fluoride membranes (PVDF, Millipore, Germany). After they were blocked with 5% fat-free milk, the membranes were incubated with primary antibodies at 4 °C overnight, followed by incubation with HRP-conjugated secondary antibodies at 37 °C for 1 h. The immunoreactive bands were visualized by using an ECL kit (Beyotime Technology, China). The primary antibodies targeted CD9 (1:1000; CST, USA, 13174 s), CD63 (1:1000; Abcam, UK, ab134045), TSG101 (1:1000; Abcam, UK, ab83), Lamin A/C (1:1000; Abcam, UK, ab108595), GM130 (1:1000; Abcam, UK, ab169276), Spred1 (1:1000; Abcam, UK, ab271191), ERK1/2 (1:1000; Abcam, UK, ab184699), p-ERK1/2 (1:1000; Abcam, UK, ab201015), PIK3R2 (1:1000; Abcam, UK, ab180967), AKT (1:1000; CST, USA, 9272 s), p-AKT (1:1000; CST, USA, 9271 s), and GAPDH (1:1000; Abcam, UK, ab181602).

### Preparation and characterization of piGEL and piGEL-sEVs

The piGEL was synthesized according to our previous reports [14]. A hydrogel precursor solution composed of o-nitrobenzyl alcohol moiety-modified hyaluronate acids (HA-NB) and gelatin (Sigma-Aldrich, USA) were mixed with Dulbecco’s PBS (DPBS) under sterile conditions. The hydrogel precursor solution was shaken at 37 °C, adjusted to pH = 7.4, and filtered through 0.22 µm sterile filters (Millipore, Germany) to obtain the liquid piGEL (content of HA-NB and gelatin was 50 mg mL^−1^ with the mass ratio of 1:1). hUSC-sEVs were mixed with the liquid piGEL at a ratio of 1:3. After the mixture was added to the surface of the vaginal mucosa defect, the tissue was irradiated with 395 nm UV light (50 mW cm^−2^) for 30 s and the light lamp was located about 5 cm above the surgical site.

### sEVs release assay

hUSC-sEVs were added to the liquid piGEL at a ratio of 1:3 to achieve a concentration of 1 × 10^10^ particles/mL. piGEL-sEVs (1000 µL) were obtained after chemical crosslinking via UV irradiation at 395 nm. Briefly, the piGEL-sEVs were immersed in PBS (1000 µL) in 24-well plates. The sEVs release profiles from the piGEL were measured using a nanoflow cytometer.

### piGEL biocompatibility assay

The biocompatibility of the piGEL was measured by live/dead cell staining. VK2 cells were seeded on piGEL in 48-well culture plates. After 24 h of incubation, the viability of VK2 cells was measured using a live/dead staining kit (Beyotime Technology, China) according to the manufacturer’s instructions. The images were observed under a fluorescence microscope (Leica DM6B, Germany).

### Establishment of vaginoplasty model

All animal experiments were approved by the Animal Research Committee of Shanghai Jiao Tong University Affiliated Sixth People’s Hospital [SYXK (Shanghai, China) 2016-0020]. Multiparous female New Zealand rabbits (30–40 weeks of age), which have proven to be a suitable model for vaginal surgery, were used in this study [20]. These rabbits were anaesthetized via ear vein administration of 20–30 mg/kg sodium pentobarbital before the operation. After shaving, vaginal mucosa defects (1 cm × 1 cm in diameter) were created under aseptic conditions. Forty rabbits were randomly divided into four treatment groups (for each group *n* = 10): (1) PBS group (100 µL of PBS); (2) piGEL group (100 µL of piGEL); (3) sEVs group (100 µL of PBS containing 10 × 10^8^ hUSC-sEVs); and (4) piGEL-sEVs group (100 µL of piGEL loaded with 10 × 10^8^ hUSC-sEVs).

At 7 or 14 days after surgery, the rabbits were sacrificed, and whole vaginal tissues were harvested (for each time point *n* = 5). Then, the vaginal mucosa defects of each group were photographed. The area of each wound was analyzed by ImageJ software (National Institutes of Health, USA).

### Histological and immunofluorescence analysis

The collected tissues were fixed in 4% paraformaldehyde (PFA, Servicebio, China), dehydrated with gradient alcohol series and embedded in paraffin. The embedded samples were cut into 6-µm-thick sections for hematoxylin and eosin (H&E) staining and Masson’s trichrome staining. Immunofluorescence (IF) staining was carried out as previously described [18]. The collected samples were fixed in 4% PFA, dehydrated with gradient series of sucrose and embedded in OCT. Ten-micrometer thick sections were incubated with primary antibodies, including those targeting AE1/AE3 (1:100; Thermo Fisher, USA, 53-9003-82), CD31 (1:100; Novus, Germany, JC/70A), and α-SMA (1:200; Abcam, UK, ab7817). Then, the sections were incubated with secondary antibodies at room temperature for 1 h. Fluorescence images were clearly observed under a Leica DM6B microscope.

### sEVs uptake by VK2 cells

To determine whether hUSC-sEVs could be internalized by VK2 cells, sEVs were labeled with Dil (Thermo Fisher, USA) and then incubated with VK2 cells at 37 °C for 12 h. Then, the cells were fixed with 4% PFA. The cytoskeletal components were stained with Actin-Tracker Green (1:100; Beyotime Technology, China), whereas the nuclei of VK2 cells were stained with DAPI (Servicebio, China). The VK2 cells were visualized and imaged under a Leica DM6B microscope.

### Cell viability and proliferation assays

The effects of hUSC-sEVs on the viability of VK2 cells were measured by CCK-8 assays. VK2 cells were seeded on 96-well plates at 3000 cells/well. At the indicated time, CCK-8 solution (Dojindo, Japan) was added to each well and cultural for another 2 h, at which point the absorbance was quantified at a wavelength of 450 nm using a microplate reader (Bio-Rad, USA). In addition, the effects of hUSC-sEVs on cell proliferation were measured by 5-ethynyl-2′-deoxyuridine (EdU) assays. After stimulation with hUSC-sEVs, VK2 cells were stained using an EdU-Click 488 proliferation kit (Beyotime Technology, China). The images of cells observed under a Leica DM6B microscope were photographed.

### Cell migration assays

The effects of hUSC-sEVs on the migration of VK2 cells were measured by scratch assays. Briefly, VK2 cells were seeded onto Ibidi culture inserts (Ibidi GmbH, Germany). When the cells reached 100% confluence, the Ibidi culture inserts were removed, and 500 µm-wide cell-free scratches were made through the monolayer. After washing with PBS, the cells were cultured in KSFM medium in the presence or absence of hUSC-sEVs. The migration behavior of cells was observed and imaged under a Leica DM6B microscope. Wound closure was calculated as follows: Migration area (%) = (*A*_0_ − *A*_n_)/*A*_0_ × 100, where *A*_0_ represents the initial wound area and A_n_ represents the remaining wound area at the measured time point.

### MicroRNA sequencing

For exosomal miRNA sequencing, total RNA was extracted and purified from hUSC-sEVs via an exoRNeasy Maxi Kit (Qiagen, Germany). The Agilent Bioanalyzer 2100 system (Agilent, USA) was used to quantify RNA. The library preparations were analyzed on the illumina Hiseq platform for gene clustering and sequencing as previously described [21]. The data were obtained from Novogene Bioinformatics Technology (China).

### miRNA transfection

The miR-126-3p inhibitor and negative control inhibitor (RiboBio, China) were transferred to hUSC-sEVs by using an Exo-Fect siRNA/miRNA Transfection Reagent kit (SBI, USA) according to the manufacturer’s protocols. These treated hUSC-sEVs were used in the following experiments.

### Quantitative real-time polymerase chain reaction (qRT-PCR) analysis

Total RNA was extracted from sEVs and cells using the miRNeasy Micro Kit (Qiagen, Germany), RNA was reverse transcribed to cDNA using 4 × Reverse Transcription Master Mix (EZBioscience, USA), and qPCR was performed using SYBR Green qPCR Master Mix (EZBioscience, USA). The primers (Sangon Biotech, China) used in this study are listed in Additional file 1: Table S1.

For miRNA analysis, exosomal miRNAs were isolated by using a miRNeasy Micro Kit (Qiagen, Germany), and cDNA for miRNAs was synthesized using miRNA cDNA 1st strand synthesis (Accurate Biotechnology, China). qRT-PCR was performed using a SYBR Green Premix Kit (Accurate Biotechnology, China), which provides miRNA reverse primers. The miRNA-specific forward primers (Sangon Biotech, China) are listed in Additional file 1: Table S2. Glyceraldehyde 3-phosphate dehydrogenase (GAPDH) and small U6 RNA were used as internal references for mRNA and miRNA, respectively.

### Statistical analysis

Each experiment in this study was performed at least three times. The results are presented as the means ± standard deviation (SD). Comparisons involving two groups were conducted with the unpaired Student’s t test whereas comparisons involving multiple groups were conducted with one-way analysis of variance (ANOVA). Statistical analysis was performed by GraphPad Prism 6.0 software. A P value < 0.05 was used to indicate statistical significance.

## Results

### Identification of hUSCs and hUSC-sEVs

**Fig. 1:**
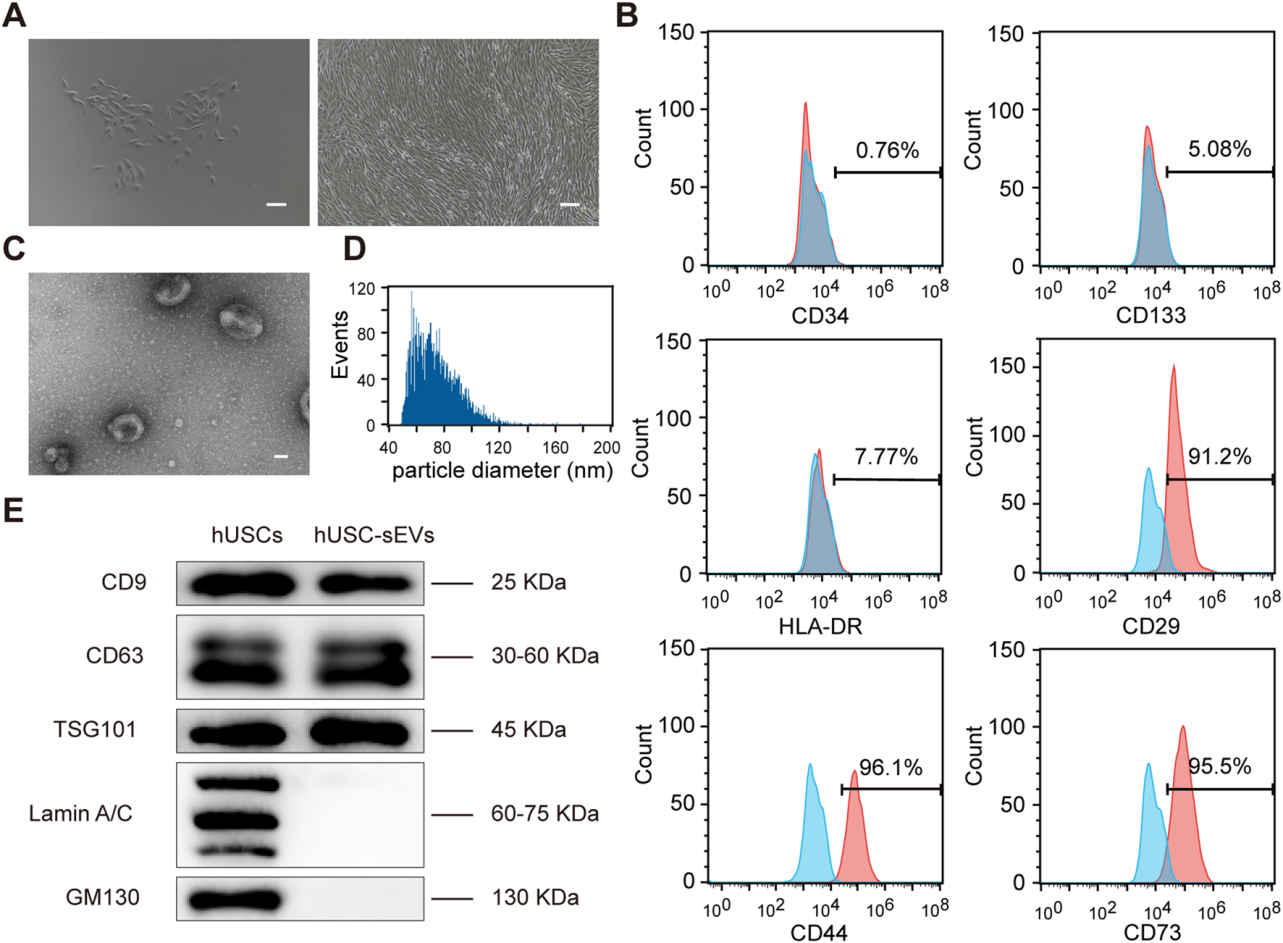
Identification of hUSCs and hUSC-sEVs. **A** Representative images of hUSCs observed by light microscopy. Scale bar: 100 µm. **B** Characteristic surface markers of hUSCs evaluated by flow cytometry. **C** Representative images of hUSC-sEVs under TEM. Scale bar: 50 nm. **D** Particle size distribution of hUSC-sEVs measured by a nanoflow cytometer. **E** Marker proteins of hUSC-sEVs were identified by western blot

Cell colonies were observed to be attached to the bottom of the plates approximately 7 days after initial seeding. Figure 1A shows that the morphology of hUSCs exhibited a fibroblast-like structure under a light microscope. Moreover, flow cytometric assays indicated that the cells were positive for CD29, CD44, and CD73 antigen and negative for CD34, CD133, and HLA-DR antigen on the surface (Fig. 1B). Thus, the results revealed that the characteristics of hUSC-sEVs were similar to those of MSCs.

TEM assays demonstrated that hUSC-sEVs were spherical microvesicles (Fig. 1C). Nanoflow assays showed the most hUSC-sEVs were approximately 50–180 nm in diameter and that the concentration of hUSC-sEVs was approximately 3 × 10^8^ particles/mL (Fig. 1D). Western blot analysis revealed that hUSC-sEVs were positive for CD9, CD63, and TSG101 but negative for Lamin A/C and GM130. (Fig. 1E). In summary, hUSC-sEVs were successfully isolated from conditioned medium of hUSCs.

### Characterization of piGEL and piGEL-sEVs

**Fig. 2:**
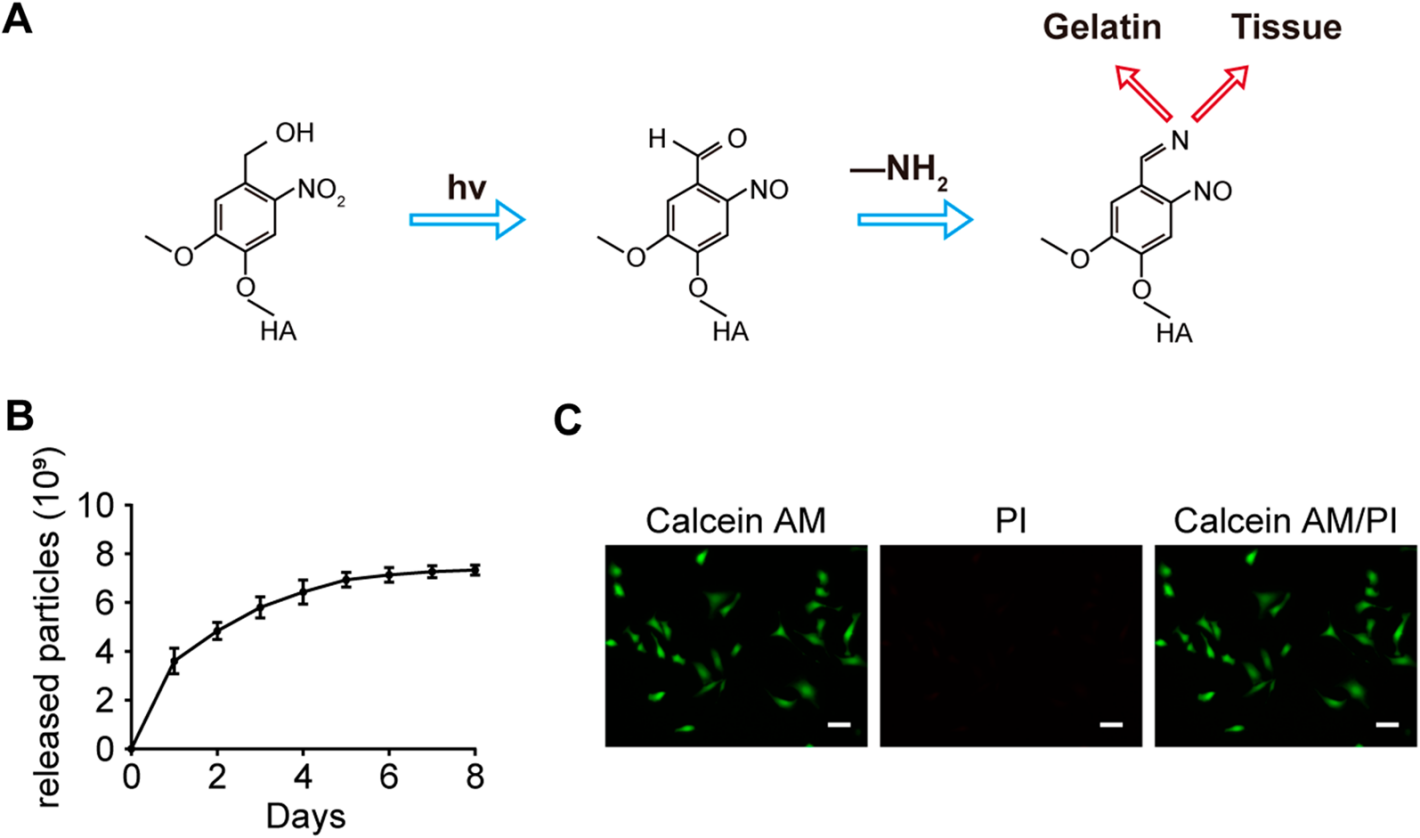
Characterization of piGEL and piGEL-sEVs. **A** Schematic diagram of the phototriggered imine crosslinking (PIC) mechanism for the integration of hydrogels and tissue. **B** Curve of release nanoparticles of piGEL-sEVs over 8 days. **C** Live/dead staining of VK2 cells cultured on piGEL. Scale bar: 100 µm

As shown in Fig. 2A, under UV irradiation at 395 nm, the mixture comprising piGEL and hUSC-sEVs rapidly solidified in situ and strongly integrated with the surface of the tissue. This process was applied in subsequent experiments. To realize the long-term sustainability of the therapeutic effect on vaginal mucosa defects, the release rate of sEVs in piGEL-sEVs is crucially important. As shown in Fig. 2B, the piGEL possessed the characteristics of slow and continuous release of sEVs. As shown in Fig. 2C, the green fluorescence represents living cells stained with calcein AM, and the red fluorescence represents dead cells stained with PI. Live/dead staining results showed that the majority of VK2 cells could maintain their viability when in contact with the piGEL, which demonstrated that the piGEL had good biocompatibility and no cytotoxicity.

### piGEL-sEVs promoted vaginal mucosa defect healing and epithelization in rabbits

**Fig. 3:**
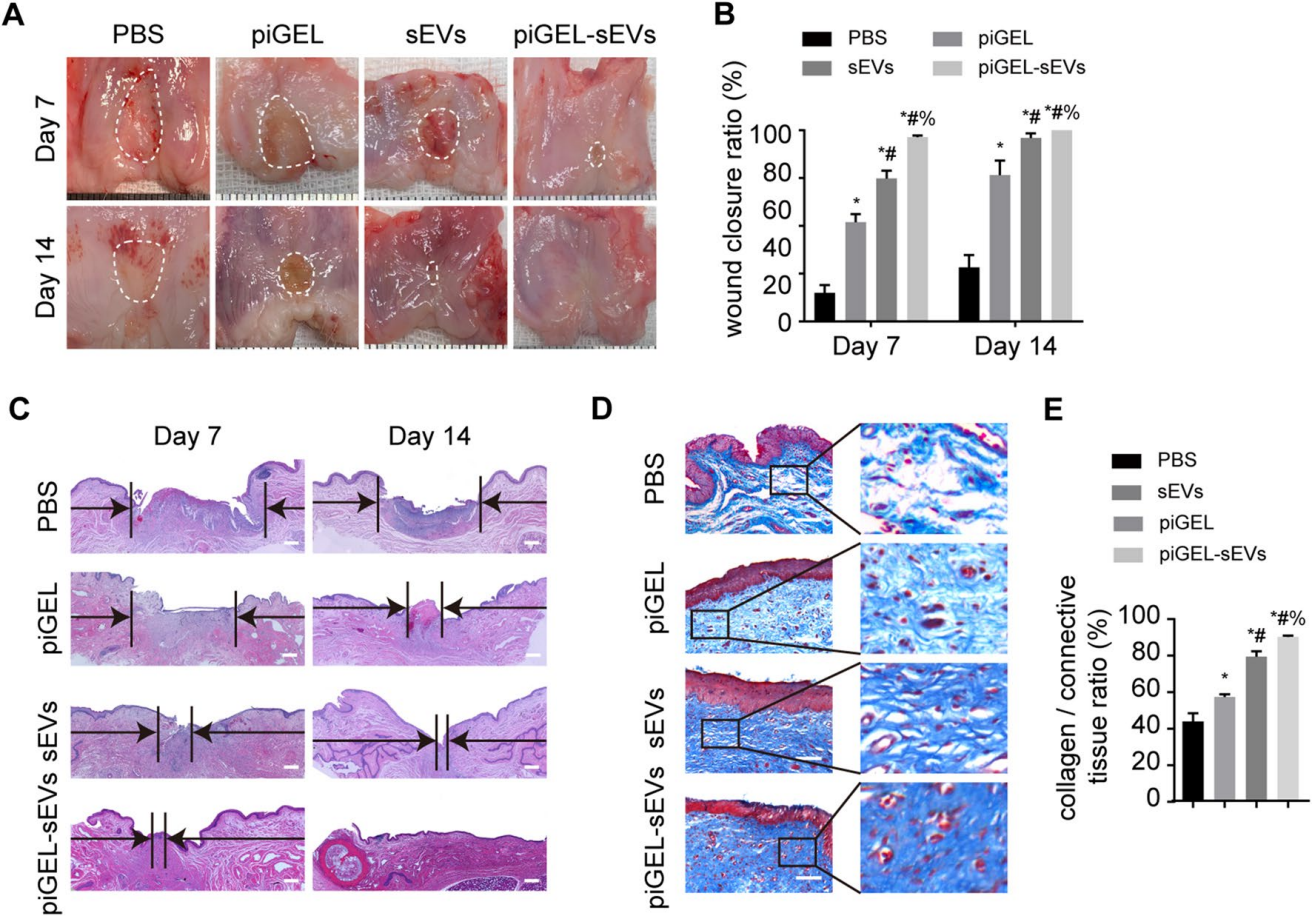
Gross and histological evaluation of vaginal mucosa defects in each group. **A** Macroscopic observation of vaginal mucosa defects at days 7 and 14 after the operation. **B** Quantitative analysis of the wound closure ratio in each group. *n* = 5 per group. **C** H&E staining of repaired vaginal wall tissue in each group. Scale bar: 500 µm. **D** Masson staining of repaired vaginal wall tissue in each group. Scale bar: 100 µm. **E** Quantitative analysis of collagen area normalized to the connective area. *, *P* < 0.05, compared with the PBS group; #, *P* < 0.05, compared with the piGEL group; %, *P* < 0.05, compared with the sEVs group

To optimize of the application of piGEL-sEVs with respect to vaginal epithelization, a vaginoplasty rabbit model was created, after which the rabbits received local administration of PBS, piGEL, sEVs, and piGEL-sEVs. As shown in Fig. 3A, the vaginal mucosa defects treated with piGEL-sEVs had almost closed at day 7, whereas large wound defects remained detectable in the PBS group. There was a significant difference in the wound closure rate between the piGEL-sEVs group and PBS group (Fig. 3B). Compared with PBS administration, piGEL, sEVs, and piGEL-sEVs administration shortened the wound healing time, indicating that piGEL-sEVs possessed a favorable stimulatory effect for vaginal tissue regeneration.

For the histological evaluation, H&E staining was performed to assess the regeneration of the newly formed tissue. H&E staining revealed that piGEL-sEVs significantly enhanced the epithelization ability of the vagina (Fig. 3C). The piGEL-sEVs group showed more intact new epidermis at day 7, which was almost similar to normal vaginal tissue by day 14 after the procedure. By contrast, there was incomplete epithelium coverage and many inflammatory cells present the regions of the vaginal mucosa defects of the PBS group at day 14. Next, we performed Masson’s staining to assess collagen formation. The collagen deposition evaluated by Masson staining in the piGEL-sEVs group increased compared to that in the PBS, piGEL, and sEVs groups (Fig. 3D, E).

Collectively, these results indicated that local piGEL-sEVs treatment accelerated wound healing and epithelization in rabbits.

### piGEL-sEVs promoted epithelium regeneration and angiogenesis of the vaginal mucosa defects in rabbits

**Fig. 4:**
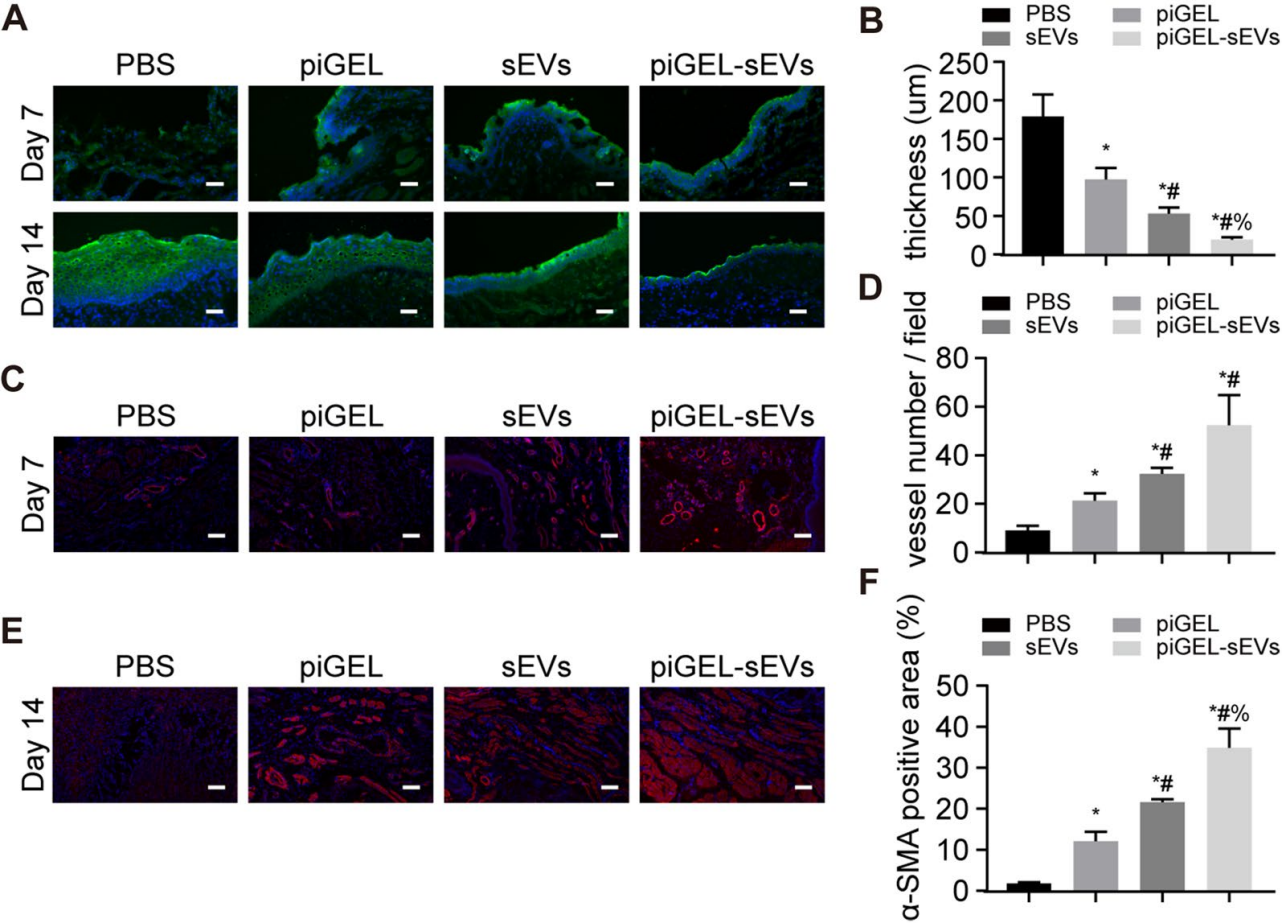
piGEL-sEVs promoted vaginal epithelium regeneration and angiogenesis. **A** IF staining for AE1/AE3 at days 7 and 14 after the operation. Scale bar: 50 μm. **B** Quantitative analysis of IF staining of AE1/AE3. *n* = 3 per group. **C** IF staining for CD31 at day 7 after the operation. Scale bar: 50 μm. **D** Quantitative analysis of new vessel number. *n* = 3 per group. **E** IF staining for *α*-SMA at day 14 after the operation. Scale bar: 50 μm. **F** Quantitative analysis of IF staining of *α*-SMA. *n* = 3 per group. *, *P* < 0.05, compared with the PBS group; #, P < 0.05, compared with the piGEL group; %, *P* < 0.05, compared with the sEVs group

The formation of epithelial structure is essential for high-quality recovery from vaginal mucosa defects [22]. IF staining of AE1/AE3 was performed to assess nascent epithelium. Within 14 days, all groups showed complete epithelization (Fig. 4A). However, the piGEL-sEVs group at day 14 showed the thinnest epithelial thickness and neatly arranged epithelial cells, which were closest to the normal vaginal epithelium (Fig. 4B, Additional file 2: Fig. S1). The PBS group presented increased epithelial thickness and disorganized epithelial cells. Angiogenesis is considered to be curial for tissue regeneration because blood vessels can transport nutrients and oxygen to the surrounding cells. Next, IF staining of CD31 was carried out to evaluate the angiogenic effects of piGEL-sEVs administration. As shown in Fig. 4C, D, there was a significant increase in the number of new vessels at day 7 in the piGEL-sEVs group, whereas fewer new blood vessels appeared in the PBS group. IF staining of *α*-SMA indicated that the levels of newly synthesized smooth muscle was significantly increased in the region with the vaginal mucosa defect at day 14 (Fig. 4E, F).

### hUSC-sEVs promoted the migration and differentiation of VK2 cells

**Fig. 5:**
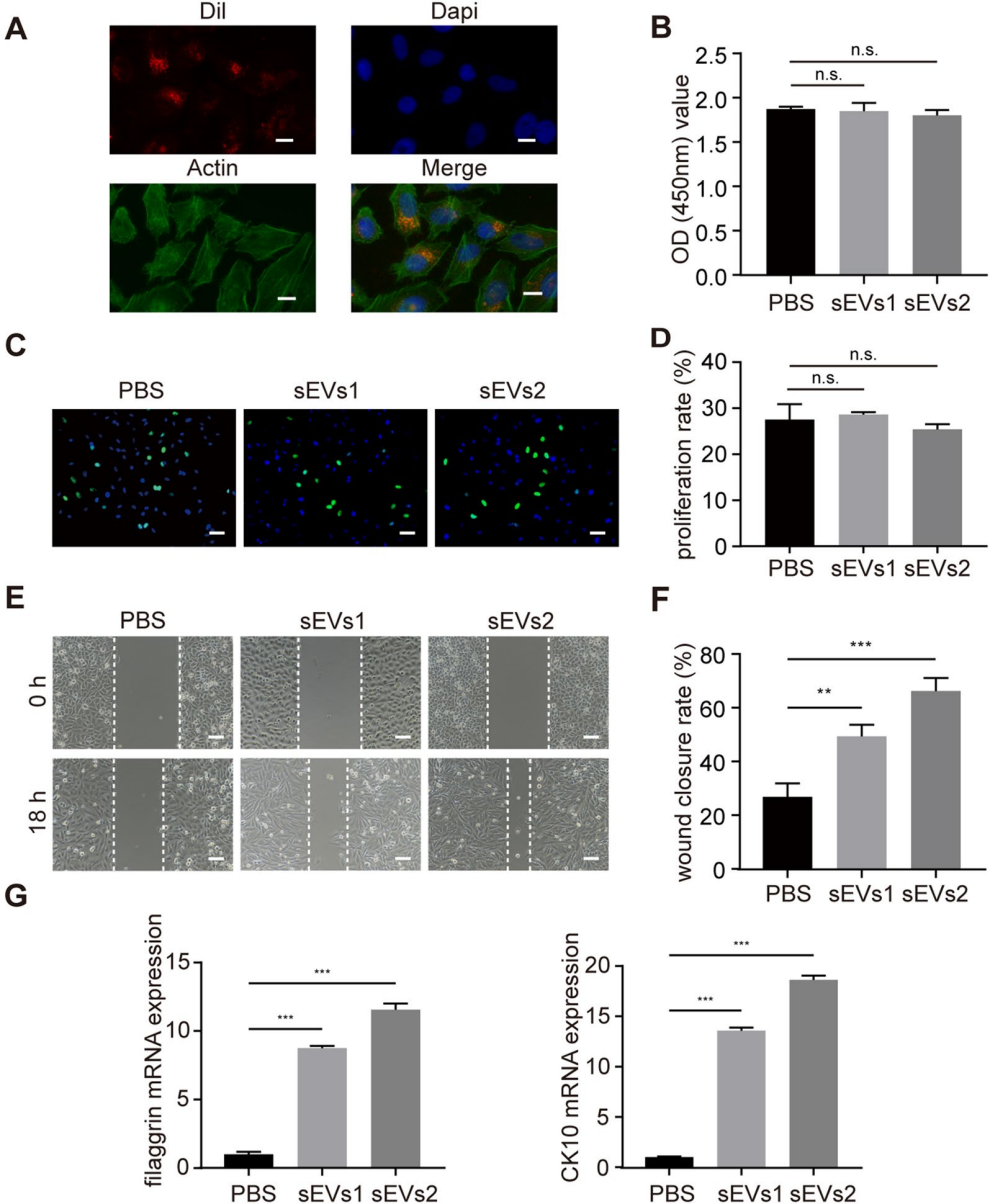
hUSC-sEVs promoted the migration and differentiation of VK2 cells. **A** Representative fluorescence micrograph of Dil (red)-labeled sEVs internalized by VK2 cells. Scale bar: 15 µm. **B** hUSC-sEVs did not increase the viability of VK2 cells at 24 h after coincubation, as detected by CCK-8 tests. **C** hUSC-sEVs did not promote the proliferation of VK2 cells at 24 h after coincubation, as detected by EdU tests. Scale bar: 50 µm. **D** Quantitative analysis of the proliferation rate of VK2 cells after treatment with hUSC-sEVs. *n* = 3 per group. **E** hUSC-sEVs promoted the migration of VK2 cells, as detected by scratch assays. Scale bar: 100 µm. **F** Quantitative analysis of the migration ability of VK2 cells after treatment with hUSC-sEVs. *n* = 3 per group. **G** mRNA expression levels of filaggrin and CK10 in VK2 cells were measured by qPCR. *n* = 3 per group. The PBS, sEVs1 and sEVs2 groups indicated 0, 5 and 10 × 10^8^ hUSC-sEVs particles/mL, respectively. *P < 0.05, **P < 0.01, ***P < 0.001, n.s. indicated nonsignificant difference

We next examined whether hUSC-sEVs had effects on the proliferation, migration and differentiation of VK2 cells in vitro. As shown in Fig. 5A, a considerable amount of Dil-labeled sEVs was in the cytoplasm of VK2 cells and around the central nucleus, which suggested that hUSC-sEVs could be internalized by recipient cells. Different concentrations of hUSC-sEVs (sEVs1 and sEVs2 at densities of 5 × 10^8^ and 10 × 10^8^ particles/mL, respectively) were coincubated with VK2 cells. CCK-8 assays were performed to evaluate the viability of VK2 cells. As shown in Fig. 5B, there was no difference in cell viability among the different treatment groups. The proliferation ability of hUSC-sEVs was further tested by EdU assays. Consistent with the CCK-8 assay results, the hUSC-sEVs treatment group had no significant promotion of VK2 cell proliferation compared to that of the PBS group (Fig. 5C, D). However, the migration ability of VK2 cells after sEVs stimulation was significantly increased compared to that of cells in the PBS group (Fig. 5E, F). Moreover, the sEVs2 group (10 × 10^8^ particles/mL), which was the highest concentration, possessed the highest migration rate.

Then, we examined the effect of hUSC-sEVs on promoting the differentiation of VK2 cells by qRT-PCR. As shown in Fig. 5G, the expression levels of epidermal differentiation markers (filaggrin, CK10) were significantly upregulated after stimulation with hUSC-sEVs compared to the PBS group. In conclusion, these results suggest that hUSC-sEVs can promote VK2 cell migration and differentiation in a concentration-dependent manner but have no effect on proliferation.

### hUSC-sEVs activated the ERK1/2 and AKT signaling pathways by delivering miR-126-3p

**Fig. 6:**
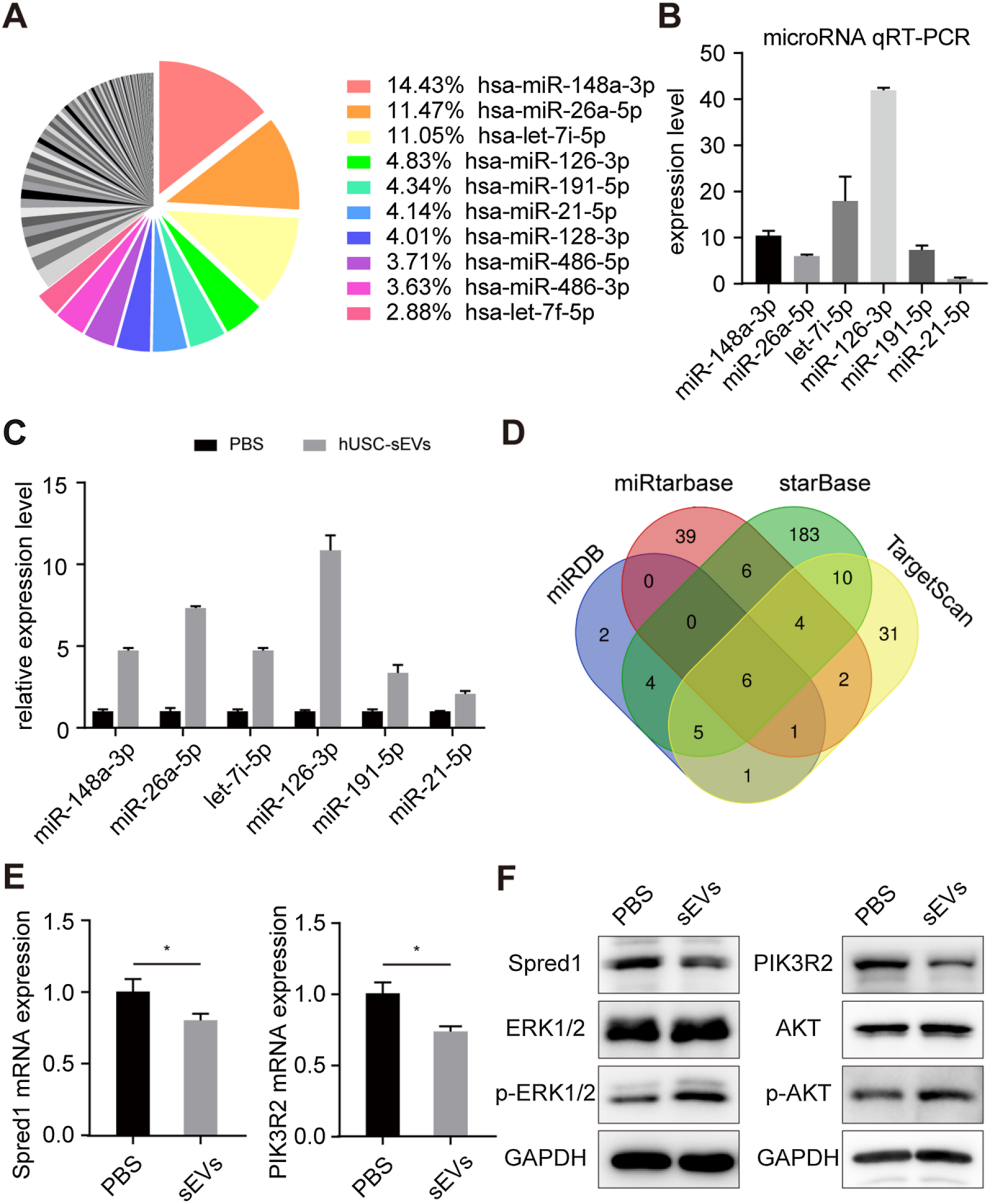
hUSC-sEVs activated the ERK1/2 and PI3K/AKT signaling pathways by delivering miR-126-3p. **A** Normalized miRNA expression levels of hUSC-sEVs measured by miRNA microarray. **B** The predicted miRNA expression levels of hUSC-sEVs measured by qPCR. **C** VK2 cells were treated with hUSC-sEVs for 6 h, and the expression of the predicted miRNAs was measured by qPCR. **D** The potential target genes of miR-126-3p were predicted by bioinformatics analysis. **E** VK2 cells were transfected with miR-126-3p for 36 h, and then the mRNA expression of Spred1 and PIK3R2 in VK2 cells was measured by qPCR. **F** VK2 cells were treated with hUSC-sEVs for 36 h, and the protein levels of Spred1, ERK1/2, p-ERK1/2, PIK3R2, AKT, and p-AKT in VK2 cells were analyzed by western blotting. The PBS and sEVs groups indicated 0 and 10 × 10^8^ hUSC-sEVs particles/mL. *P < 0.05, **P < 0.01, ***P < 0.001

It has been shown that sEVs may play an important role in tissue regeneration by transferring miRNAs to recipient cells and then regulating the expression of related genes. To investigate the miRNA expression levels in hUSC-sEVs, we carried out a microarray analysis. As shown in Fig. 6A, a large number of miRNAs were detected in hUSC-sEVs. The top 6 most abundant miRNAs were miR-148a-3p, miR-26a-5p, let-7i-5p, miR-126-3p, miR-191-5p, and miR-21-5p, which comprised approximately 50% of all the miRNAs. Moreover, the expression of the top 6 miRNAs was examined by qRT-PCR assays, which revealed that miR-126-3p was ranked highest among various miRNAs, indicating that hUSC-sEVs might play a critical role in performing their biological function by delivering miR-126-3p (Fig. 6B). To determine the effect of miRNA transfer, we explored the change in miRNA expression in VK2 cells after stimulation with hUSC-sEVs. As shown in Fig. 6C, the expression of miR-126-3p in VK2 cells resulted in the greatest growth among the top 6 miRNAs after stimulation with hUSC-sEVs for 6 h. Thus, we focused on exosomal miR-126-3p for further investigation.

Subsequently, we predicted the potential target genes of miR-126-3p the miRDB, miRtarbase, starBase and TargetScan database (Fig. 6D). Among the predicted target genes, Spred1 and PIK3R2 have been reported to be related to migration and differentiation. Many studies have demonstrated that Spred1 and PIK3R2 are direct target genes of miR-126-3p and can be suppressed by miR-126-3p [23–25]. As shown in Fig. 6E, the protein and mRNA expression levels of Spred1 and PIK3R2 were markedly reduced following treatment with hUSC-sEVs for 36 h. Collectively, the results indicated that exosomal miR-126-3p could be transferred into VK2 cells to regulate the expression of Spred1 and PIK3R2. Western blotting analysis revealed that hUSC-sEVs treatment upregulated ERK1/2 phosphorylation (p-ERK1/2) and AKT phosphorylation (p-AKT) (Fig. 6F). Thus, we suggested that the underlying mechanism of hUSC-sEVs on migration and differentiation was the activation of the ERK1/2 and AKT signaling pathways. These results indicated that hUSC-sEVs could deliver miR-126-3p to inhibit Spred1 and PIK3R2 expression and then activate the corresponding downstream signaling pathways.

### miR-126-3p inhibitor attenuated the effects of hUSC-sEVs on VK2 cells in vitro

**Fig. 7:**
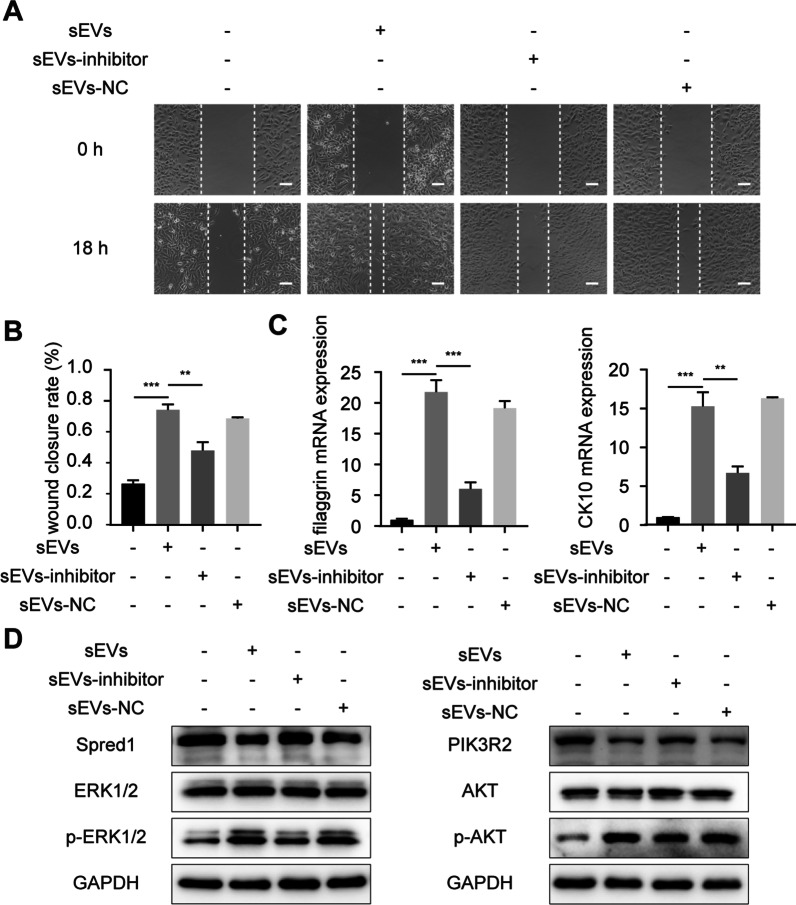
miR-126-3p silencing attenuated the effect of hUSC-sEVs on VK2 cells. **A** Representative images of the scratch migration assay of VK2 cells. Scale bar: 100 µm. **B** Quantitative analysis of the migration behavior of VK2 cells. *n* = 3 per group. **C** VK2 cells were treated with miR-126-3p inhibitor or control inhibitor for 36 h, and the expression of filaggrin and CK10 was measured by qPCR. *n* = 3 per group. **D** The protein levels of Spred1, ERK1/2, p-ERK1/2, PIK3R2, AKT and p-AKT were analyzed by western blotting. **E** Densitometric quantification of the relative band intensity in **D**. *n* = 3 per group. *P < 0.05, **P < 0.01, ***P < 0.001

To further investigate the role of miR-126-3p, we next evaluated whether it may affect the promotion of migration and differentiation caused by hUSC-sEVs stimulation. First, we transfected hUSC-sEVs with an miR-126-3p inhibitor to silence miR-126-3p expression in hUSC-sEVs. As shown in Fig. 7A, B, the hUSC-sEVs-induced migratory effect was partially reduced by the presence of an miR-126-3p inhibitor. In addition, qRT-PCR was performed to measure the effect of miR-126-3p on the differentiation ability of VK2 cells. As shown in Fig. 7C, the miR-126-3p inhibitor significantly impaired the differentiation ability mediated by hUSC-sEVs in VK2 cells compared to that in the sEVs group. Western blotting analysis showed that the protein expression of Spred1 and PIK3R2, which are target genes of miR-126-3p, was increased by miR-126-3p inhibitor treatment (Fig. 7D, E). Subsequently, we investigated the activity of the ERK1/2 and AKT signaling pathways in VK2 cells after miR-126-3p inhibitor administration. The protein expression of p-ERK1/2 and p-AKT in VK2 cells was decreased in the miR-126-3p inhibitor group, indicating that inhibition of miR-126-3p markedly influenced the ERK1/2 and AKT signaling pathways activated by hUSC-sEVs. Together, these results indicated that the positive effects of hUSC-sEVs on VK2 cells are mediated by miR-126-3p-induced inhibition of Spred1 and PIK3R2 expression.

## Discussion

To prolong the retention time of sEVs on the surface of the wound, we packaged hUSC-sEVs into piGEL, which could ensure the sustained release of sEVs during the healing period. The in vivo experiments indicated that piGEL-sEVs effectively accelerated epithelization of vaginal mucosa defects after partial vaginectomy. In vitro, we found that hUSC-sEVs could effectively strengthen the migration and differentiation of vaginal epithelial cells by delivering miR-126-3p to VK2 cells. In addition, we observed that Spred1 and PIK3R2 (target genes of miR-126-3p) and the ERK1/2 and AKT signaling pathways participated in this process. These results demonstrated that hUSC-sEVs-based miRNA-126-3p gene therapy is a promising approach to enhance the epithelization of neovagina after vaginoplasty.

The ideal treatment after vaginoplasty requires a smooth, elastic and moist vaginal canal with adequate diameter and length. Currently, various treatments for surgical neovaginal reconstruction have been reported, such as autologous tissue and bioengineering materials, including epidermal tissue grafts [26], oral mucosa [27], in vitro cultured vaginal tissue [28], acellular dermal matrix [29], and acellular porcine small intestinal submucosa [30]. Nevertheless, the clinical applications of these treatments are restricted because of graft contracture, poor fixation, limited resources, increased donor site morbidity, graft rejection reaction and so on. Moreover, there are many postoperative complications, such as vaginal stenosis, necrosis, shrinkage, and scar formation [31].

**Fig. 8:**
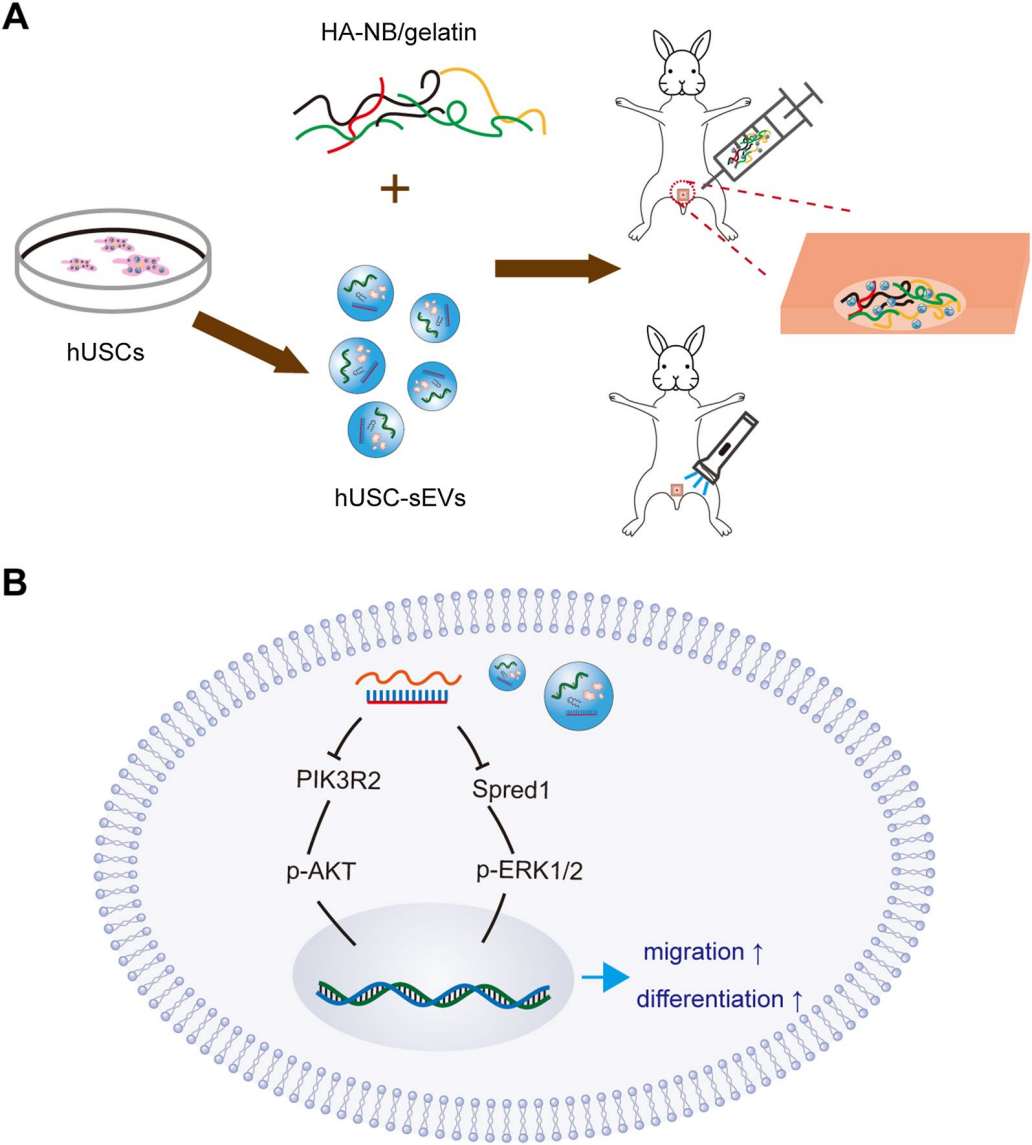
Schematic diagram of therapeutic sEVs released from piGEL for epithelization of the vagina. **A** Fabrication and application of piGEL-sEVs. **B** The underlying mechanisms of hUSC-sEVs on VK2 cells

Dressings play crucial roles in wound management because they can protect the wound from external infection and accelerate the healing process. Hydrogels elicit great tissue regeneration potential due to their high biocompatibility, good degradability and characteristics similar to extracellular matrix materials. In our previous study, a novel injectable hydrogel with good tissue adhesion and self-adapting features was developed [14]. Under light irradiation, the aldehyde group was crosslinked with the amino group of piGEL while also reactive with the amino group of the surface of the tissue. Thus, piGEL-sEVs were strongly integrated with the tissue via covalent bonding and physical adhesion. After vaginoplasty, the size, shape and thickness of vaginal mucosa defects are usually irregular. Therefore, piGEL in situ molding materials that adapt well to varying conditions are desirable for vaginal mucosa defects. Moreover, due to its three-dimensional structure, piGELs are a suitable choice as sEVs delivery carriers, providing a stable and desirable microenvironment for cell proliferation and migration and differentiation. In this study, we successfully fabricated piGEL-sEVs. In a rabbit model of partial vaginectomy, piGEL-sEVs enriched with miR-126-3p could be gradually released while ensuring a long stay in the tissue surface. The absorbed hUSC-sEVs were internalized by vaginal cells and accelerated the morphologic and functional recovery of vaginal mucosa defects (Fig. 8A).

Stem cells, a new therapeutic option for regenerative medicine, have been demonstrated to accelerate epithelization of wounds, although the molecular mechanisms remain unknown [32]. Compared with MSCs, autologous hUSCs have more clinical therapeutic value for vaginal tissue repair due to their desirable biological properties such as abundant sources, painless accession, easy collection, and low immunogenicity in vitro [33–35]. sEVs have been shown to have a similar function to that of parent cells by carrying the same biological factors [36]. In our previous studies, we found that hUSC-sEVs have promising therapeutic effects in wound healing and neurogenesis [15, 16, 37, 38]. In this study, we found that hUSC-sEVs could be successfully internalized by VK2 cells, which then promoted the migration and differentiation of VK2 cells in vitro. Nevertheless, the role of hUSC-sEVs in vaginal regeneration remains unclear, and the underlying mechanism remains to be researched.

Accumulating evidence suggests that exosomal miRNAs may regulate recipient cell activity by degrading or repressing the translation of target mRNAs. Some studies have reported that the growth of VK2 cells could be regulated via miRNAs of sEVs derived from UC-MSCs, suggesting that exosome-mediated transfer of miRNA exhibits great potential in vaginal tissue repair [39]. Therefore, we detected the miRNAs in hUSC-sEVs and found that miR-126-3p was the most highly expressed miRNA among the detected miRNAs. It is widely accepted that miR-126 plays an essential regulatory role in physiological angiogenesis and vascular integrity [40]. The underlying mechanisms may involve targeting the genes Spred1 and PIK3R2, which are negative regulators of the ERK1/2 and AKT signaling pathways. The ERK1/2 and AKT signaling pathways are closely associated with cell proliferation, migration, differentiation and angiogenesis [41, 42]. Therefore, miRNA-126-3p is a promising candidate for tissue regeneration. The underlying mechanism revealed that hUSC-sEVs delivered miR-126-3p to VK2 cells and that miR-126-3p downregulated the expression of Spred1 and PIK3R2, resulting in the activation of the ERK1/2 and AKT pathways (Fig. 8B). In this study, when the expression of miR-126-3p in hUSC-sEVs was downregulated, the effects of hUSC-sEVs on migration and differentiation were decreased. Further studies demonstrated that the miR-126-3p inhibitor failed to downregulate the expression of Spred1 and PIK3R2, and activate the ERK1/2 and AKT signaling pathways. Nevertheless, the positive effect of hUSC-sEVs was partially attenuated by the miR-126-3p inhibitor, suggesting that other important molecules of hUSC-sEVs may be involved in the treatment process.

## Conclusion

In summary, our results demonstrated that hUSC-sEVs promoted the migration and proliferation of VK2 cells by delivering miR-126-3p and that the designed piGEL-sEVs significantly accelerated vaginal mucosa defect healing and epithelialization. Taken together, the data from our study could provide a novel and promising approach for enhancing epithelization of the neovagina after vaginoplasty.

## Supplementary Information

**Additional file 1: Table S1.** The PCR primers used in this study. **Table S2.** The miRNA-specific forward primers used in this study.

**Additional file 2: Fig. S1.** Histology of normal vaginal tissue. **A** IF staining for AE1/AE3. Scale bar: 25 µm. **B** The thickness of epithelium in normal vagina was 19.75 ± 5.23 µm.

## Data Availability

The datasets used and/or analyzed during the current study are available from the corresponding author on reasonable request.

## Abbreviations

MRKH: Mayer–Rokitansky–Küster–Hauser
MSCs: Mesenchymal stem cells
UC-MSCs: Umbilical cord MSCs
hUSCs: Human urine-derived stem cells
sEVs: Small extracellular vesicles
hUSC-sEVs: Small extracellular vesicles derived from human urine-derived stem cells
miRNAs: MicroRNAs
piGEL: A phototriggered imine crosslink hydrogel
DMEM: High-glucose Dulbecco’s modified eagle medium
FBS: Fetal bovine serum
PBS: Phosphate-buffered saline
TEM: Transmission electron microscopy
SDS-PAGE: Sodium dodecyl sulfate–polyacrylamide gel electrophoresis
PVDF: Polyvinylidene fluoride membranes
HA-NB: O-nitrobenzyl alcohol moiety-modified hyaluronate acids
PFA: Paraformaldehyde
H&E: Hematoxylin and eosin
IF: Immunofluorescence
GAPDH: Glyceraldehyde 3-phosphate dehydrogenase
