# Supplementary material for: miR-126-3p-loaded small extracellular vesicles secreted by urine-derived stem cells released from a phototriggered imine crosslink hydrogel could enhance vaginal epithelization after vaginoplasty
Source: Stem Cell Res Ther. 2022 Jul 23;13:331. doi: 10.1186/s13287-022-03003-x (PMC9308191; doi:10.1186/s13287-022-03003-x)
Supplement: Supplementary file 1 — Additional file 1: Table S1. The PCR primers used in this study. Table S2. The miRNA-specific forward primers used in this study. [file 13287_2022_3003_MOESM1_ESM.docx]

**Supplementary data**

**Table S1.** The PCR primers used in this study.

| Gene name | Forward primer (5’ ➝ 3’) | Reverse primer (5’ ➝ 3’) |
| --- | --- | --- |
| hsa-Filaggrin | TGAAGCCTATGACACCACTGA | TCCCCTACGCTTTCTTGTCCT |
| hsa-CK10 | TCCTACTTGGACAAAGTTCGGG | CCCCTGATGTGAGTTGCCA |
| hsa-Spred1 | AAGGATGCCCCGAATCAAAAA | GGCTTGGCTTTGCATGTAGAC |
| hsa-PIK3R2 | AAAGGCGGGAACAATAAGCTG | CAACGGAGCAGAAGGTGAGTG |
| hsa-GAPDH | AGCCACATCGCTCAGACAC | GCCCAATACGACCAAATCC |

**Table S2.** The miRNA-specific forward primers used in this study.

| Gene name | Forward primer (5’ ➝ 3’) |
| --- | --- |
| hsa-miR-148a-3p | CCGTCAGTGCACTACAGAACTTTGT |
| hsa-miR-26a-5p | GCGTTCAAGTAATCCAGG |
| hsa-let-7i-5p | CTGAGGTAGTAGTTTGTGCTGTT |
| hsa-miR-126-3p | TCGTACCGTGAGTAATAATGC |
| hsa-miR-191-5p | GCCGAATCCCAAAAGCAGC |
| hsa-miR-21-5p | CCCACTGACTGATGTTGAAAA |
| U6 | GGAACGATACAGAGAAGATTAGC |
