# Supplementary material for: miR-126-3p-loaded small extracellular vesicles secreted by urine-derived stem cells released from a phototriggered imine crosslink hydrogel could enhance vaginal epithelization after vaginoplasty
Source: Stem Cell Res Ther. 2022 Jul 23;13:331. doi: 10.1186/s13287-022-03003-x (PMC9308191; doi:10.1186/s13287-022-03003-x)
Supplement: Supplementary file 2 — Additional file 2: Fig. S1. Histology of normal vaginal tissue. A IF staining for AE1/AE3. Scale bar: 25 µm. B The thickness of epithelium in normal vagina was 19.75 ± 5.23 µm. [file 13287_2022_3003_MOESM2_ESM.docx]

**
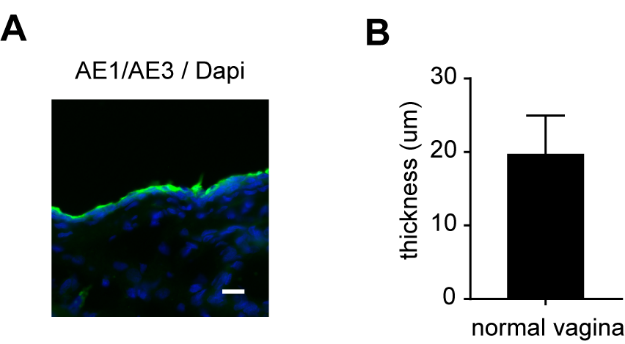
**

**Figure S1.** Histology of normal vaginal tissue. **A** IF staining for AE1/AE3. Scale bar: 25 µm. **B** The thickness of epithelium in normal vagina was 19.75 ± 5.23 µm.
